# Complement dysregulation and Alzheimer's disease in Down syndrome

**DOI:** 10.1002/alz.12799

**Published:** 2022-09-23

**Authors:** Aurora Veteleanu, Sarah Pape, Kate Davies, Eleftheria Kodosaki, Abdul Hye, Wioleta M. Zelek, Andre Strydom, B. Paul Morgan

**Affiliations:** ^1^ School of Medicine UK Dementia Research Institute Cardiff University Cardiff UK; ^2^ Department of Forensic and Neurodevelopmental Science Institute of Psychiatry Psychology and Neuroscience King's College London UK; ^3^ School of Medicine, Division of Infection and Immunity Cardiff University Cardiff UK

**Keywords:** Alzheimer's disease, complement system, dementia, Down syndrome, immune dysregulation

## Abstract

**Introduction:**

Down syndrome (DS) is associated with immune dysregulation and a high risk of early onset Alzheimer's disease (AD). Complement is a key part of innate immunity and driver of pathological inflammation, including neuroinflammation in AD. Complement dysregulation has been reported in DS; however, the pattern of dysregulation and its relationship to AD risk is unclear.

**Methods:**

Plasma levels of 14 complement biomarkers were measured in 71 adults with DS and 46 controls to identify DS‐associated dysregulation; impact of apolipoprotein E (*APOE*) ε4 genotype, single nucleotide polymorphisms (SNPs) in *CLU* and *CR1*, and dementia on complement biomarkers was assessed.

**Results:**

Plasma levels of complement activation products (TCC, iC3b), proteins (C1q, C3, C9), and regulators (C1 inhibitor, factor H, FHR4, clusterin) were significantly elevated in DS versus controls while FI and sCR1 were significantly lower. In DS with AD (*n* = 13), C3 and FI were significantly decreased compared to non‐AD DS (*n* = 58). Neither *APOE* genotype nor *CLU* SNPs impacted complement levels, while rs6656401 in *CR1* significantly impacted plasma sCR1 levels.

**Conclusions:**

Complement is dysregulated in DS, likely reflecting the generalized immune dysregulation state; measurement may help identify inflammatory events in individuals with DS. Complement biomarkers differed in DS with and without AD and may aid diagnosis and/or prediction.

**Highlights:**

- Complement is significantly dysregulated in plasma of people with DS who show changes in levels of multiple complement proteins compared to controls.
- People with DS and dementia show evidence of additional complement dysregulation with significantly lower levels of C3 and factor I compared to those without dementia.
- rs6656401 in CR1 was associated with significantly elevated sCR1 plasma levels in DS.

## 1 BACKGROUND

Down syndrome (DS), caused by triplication or partial triplication of chromosome 21, is the most common chromosomal abnormality in humans, affecting 6 million people worldwide.^1^ In addition to intellectual disability, DS predisposes individuals to autoimmune diseases and several chronic pathologies, including congenital heart defects, and ophthalmic diseases.^2^ Although advances in medicine have substantially improved outcome in recent years, these multiple pathologies impact both life expectancy and quality of life for people with DS.^3^ Life expectancy remains low, now averaging 60 to 65 years, almost 20 years below that of the general population. With increasing longevity, numerous age‐related comorbidities have been identified in DS.^4^, ^5^ By the age of 40, almost all individuals affected by DS display typical Alzheimer's disease (AD) neuropathology, notably the presence of amyloid beta (Aβ) plaques, a pathological hallmark of AD,^6^, ^7^ associated with early onset dementia.^8^ Accelerated amyloid accumulation is likely a consequence of triplication of the amyloid precursor protein (*APP*) gene located on chromosome 21, leading to increased APP expression and rapid and sustained deposition of amyloid protein in the brain.^8^ Additionally, neurofibrillary tau has been shown to accompany Aβ deposits in DS brains as early as Braak stage I.^9^

DS is now recognized as an immune dysregulation disorder, with anomalies affecting both the innate and adaptive immune systems.^10^ Children with DS frequently present with autoimmune conditions, are more likely to get severe infections, and spend more time in hospital as a consequence.^11^ Aberrant neuroinflammatory processes in DS likely contribute to the development of AD‐like pathology, neurodegeneration, and dementia.^12^

Complement, a core part of the innate immune system, comprises >30 factors in plasma and on cells; these cooperate in a complex network to recognize and eliminate a variety of pathogens. Activation on pathogens, debris, or damaged cells triggers an enzymatic cascade that generates opsonic and pro‐inflammatory products; these facilitate elimination of pathogens and debris but can, if dysregulated, also drive pathology.^13^ There is a large body of evidence implicating complement dysregulation as a driver of neuroinflammation in AD. Aggregated Aβ directly activates the complement cascade by interacting with C1q,^14^ and several studies have shown C1q immunoreactivity colocalizing with Aβ in amyloid plaques in AD brain tissue.^15^, ^16^, ^17^ Similar patterns of C1q, C3 fragments, clusterin, and activated microglia localizing to amyloid plaques were reported in brains from DS individuals as young as 15 years old.^15^, ^18^

Complement biomarker studies in AD cerebrospinal fluid (CSF) and plasma have provided additional evidence of complement dysregulation and shown that complement activation products and proteins can aid diagnosis and predict progression of AD.^19^, ^20^ Additionally, single nucleotide polymorphisms (SNPs) in *CLU* and *CR1* were significantly associated with AD risk in genome‐wide association studies (GWAS).^21^ The AD risk SNP rs6656401 in *CR1* was strongly associated with *CR1* length polymorphism, minor allele carriers expressing the longer form of CR1.^22^ To date, there have been no comprehensive studies of complement biomarkers and no analysis of the AD‐associated complement SNPs in DS. Elevated plasma C3 levels were reported in adolescents with DS compared to controls;^23^ however, other complement proteins that are informative in AD, including clusterin and soluble complement receptor 1 (sCR1),^24^, ^25^ have not been measured in DS. In this study, we have quantified 14 proteins implicated in the complement cascade, including key components (C1q, C3, C4, C9), regulators (C1 inhibitor, FI, sCR1, factor H [FH], FHR4, FHR125, clusterin), and activation products (iC3b, C5a, TCC) in plasma from individuals with DS (*n* = 71) and age‐matched healthy controls (*n* = 46). We have used these data to assess the level of complement dysregulation in DS and identify complement biomarkers that correlate with dementia status. Genetic information, available for 58 of the participants with DS, was used to assess the effects of apolipoprotein E (*APOE*) genotype and the AD‐associated SNPs rs11136000 (*CLU*), rs6656401, rs6691117 (*CR1*) on complement protein levels in DS.

## 2 METHODS

### 2.1 Ethics

Ethical approval for the London Down Syndrome Consortium (LonDownS) study was granted by the North West Wales Research Ethics Committee (13/WA/0194). Written informed consent was obtained from individuals with capacity to consent after a full explanation of the study. For individuals who lacked capacity to provide informed consent, a consultee signed on their behalf to indicate their decision regarding the individual's inclusion based on their knowledge of the individual and his/her wishes. This is in accordance with the UK Mental Capacity Act 2005.

### 2.2 Subjects

Plasma samples for complement analysis from individuals with DS (*n* = 71) were obtained from the LonDownS, a prospective longitudinal study following adults with DS.^26^ Demographic and health data including body mass index (BMI) were collected contemporaneously.

Presence of DS was confirmed genetically on blood or saliva samples. After DNA extraction, genome‐wide SNP genotyping was performed using an Illumina OmniExpressExome array v1.2‐1.4 at University College London Genomics. DNA data was run in Genome Studio and converted to the PLINK format. Data from the *CR1* region of chromosome 1 and *CLU* region of chromosome 8 was used to identify the relevant SNPs. *APOE* status was determined using TaqMan assays for rs7412 and rs429358 (Thermo Fisher Scientific). Level of intellectual disability was based on International Classification of Diseases 10th revision diagnostic criteria. Dementia diagnosis was taken from clinical records and medical history; however, subjects with dementia were not considered a separate population and were analyzed together with non‐demented individuals against controls. Plasma samples were collected in ethylenediaminetetraacetic acid (EDTA) tubes, centrifuged at 2000 × *g* at 4°C for 10 min, and stored at the Social, Genetic and Developmental Psychiatry (SGDP) Centre, King's College London at –80°C until analysis. Age‐matched control plasma samples (*n* = 46) were obtained from consented healthy volunteer donors at Newcastle University (kind gift of Prof Claire Harris; REC ref. ^12^/NE/0121). Blood was collected in EDTA tubes, centrifuged at 4°C for 10 min, and plasma collected and stored at –80°C. Neither DS nor control samples were subjected to additional freeze–thaw cycles. Demographic data are in Table 1.

**TABLE 1 alz12799-tbl-0001:** Clinical and demographic characteristics of the cohort

	Down syndrome	Control
Number	71	46
Sex, n (%)*		
Female	25 (35.7)	29 (63)
Male	45 (64.3)	17 (37)
Unknown	1	
Race, n		
White	62	
Other	6	
Unknown	3	46
Age**		
Mean (SD)	40.69 (13.22)	37 (11.7)
Range	21–69	22–65
Learning disability, n		
Mild	30	n/a
Moderate	34	n/a
Severe/profound	6	n/a
Dementia, n	13	n/a
Mean age at dementia onset (SD)	52.5 (13.3)	n/a
Mean BMI (SD)	28.8 (6.6)	n/a
*APOE* status, n		
ε4 (‐)	47	
ε4 (+)	16	
Unknown	9	46
rs11136000 genotype, n		
CC	21	
TC	25	
TT	12	
rs6656401 genotype, n		
GG	42	
AG	12	
AA	3	
rs6691117 genotype, n		
AA	33	
GA	22	
GG	3	

Abbreviations: *APOE*, apolipoprotein E; BMI, body mass index; SD, standard deviation.

### 2.3 Measurement of complement proteins by enzyme‐linked immunosorbent assay

Fourteen complement components, regulators, and activation products were measured by sandwich enzyme‐linked immunosorbent assay (ELISA) using commercial or in‐house produced antibodies. Antibodies and proteins used in the ELISA are listed in Table S1 in supporting information. Plasma samples stored at –80°C were thawed immediately prior to use in ELISA, vortexed briefly, diluted in 0.2% bovine serum albumin (BSA) in phosphate‐buffered saline containing 0.05% Tween‐20 (PBST), and kept on ice until use. Activation products (TCC, iC3b, C5a) were measured immediately after thawing to avoid in vitro complement activation, while other proteins were measured in either undiluted or prediluted samples kept on ice and measured within 24 h of thawing. Standard proteins were purified by affinity chromatography from healthy donor serum/plasma and purity confirmed by sodium dodecyl sulfate polyacrylamide gel electrophoresis. Protein concentrations were determined by bicinchoninic acid assay. In‐house monoclonal antibodies (mAbs) were produced using hybridoma technology; mAb were tested for specificity in ELISA and western blotting. Sandwich ELISAs were developed in house for quantification of complement activation products, regulators, and proteins in plasma samples; they were evaluated for sensitivity, reproducibility, and intra‐ and inter‐assay coefficients of variation (CV) in 10 healthy control plasma samples. Linearity experiments were performed for each assay to determine a suitable dilution factor for plasma samples, chosen as the dilution falling within the linear portion of the log standard curve for all controls. The assays were validated following the US Food and Drug Administration Q2(R1) Validation Guidelines.^27^ Purified protein was spiked into the protein‐depleted plasma at three known concentrations (i.e., 50, 100, and 150 μg/ml) and concentration measured in the ELISA to determine accuracy, repeatability, linearity, and range. Assay reproducibility was calculated using a published method and template.^27^ Assay performance was assessed by taking multiple measures from independently diluted aliquots of the same plasma sample set; the intra‐assay CV, calculated as described,^28^ was less than 10% and inter‐assay CV (*n* = 3) was between 1% and 14% in the different assays (Table S1).

1 **RESEARCH IN CONTEXT**

1. **Systematic review**: The authors used PubMed to search previous studies investigating the role of complement in Down Syndrome (DS). Some studies examined complement in DS and DS with Alzheimer's disease (AD) (Sullivan et al, 2017; Heinonen et al, 1993; Gutierrez‐Hervas et al, 2020), however there was a lack of studies which comprehensively analysed complement proteins in plasma from people with DS.
2. **Interpretation**: In a sample of DS subjects (n=71) from the LonDownS study we show evidence of complement dysregulation compared to healthy controls (n=46), with significantly elevated plasma levels of complement proteins (C1q, C3, C9), regulators (C1 inhibitor, clusterin, FH, FHR4) and activation markers (TCC, iC3b). Dementia due to AD in DS is associated with further changes in levels of complement proteins and regulators; C3 and factor I were significantly lower in demented vs non‐demented individuals.
3. **Future directions**: Our findings provide insight into the extent of complement dysregulation in adults with DS with and without AD. The evidence strongly supports significant complement dysregulation in classical and terminal pathways. It is essential to determine whether complement is a driver of immune‐related comorbidities in DS and whether it contributes to AD pathology in DS to better understand disease pathways and develop targeted therapies.

For each assay,[Table alz12799-tbl-0001] wells of 96‐well Maxisorp (Nunc) immunoplates (Fisher Scientific #10394751) were coated (1 h at 37°C or overnight at 4°C) with capture antibodies at concentrations between 1 and 5 μg/ml in 50 μl/well carbonate‐bicarbonate buffer (pH 9.6). Wells were then blocked by incubation with 100 μl blocking buffer (2% BSA in PBST) for 1 h at 37°C. Plates were washed once with PBST, 50 μl plasma samples (in duplicate) added at a suitable dilution in 0.2% BSA in PBST, together with the appropriate standard protein serially diluted in duplicate in the range appropriate to the assay. Assays were then incubated for 90 min at 37°C, plates washed three times, and detection antibodies added at concentrations between 1 to 5 μg/ml in 50 μl/well 0.2% BSA in PBST for 1 h at 37°C. For assays for which the detection antibody was not directly labelled, a horseradish peroxidase (HRP)‐labelled secondary antibody (anti‐mouse or anti‐rabbit IgG as appropriate, Jackson ImmunoResearch #715‐035‐151, #711‐035‐152), or for biotinylated detection antibodies, Streptavidin‐HRP (R&D systems #DY998), was added to washed plates at a suitable dilution for 1 h at 37°C. Finally, plates were washed and developed using OPD substrate (Sigma‐Aldrich, #P9187) for 3 to 15 min (development time consistent throughout for each assay), followed by the addition of 5% H_2_SO_4_ to stop the reaction. Optical densities were read at 492 nm using a microplate reader (Infinite F50, Tecan #30190077). When a sample qualified as an outlier (1.5 times higher than the third quartile or 1.5 times lower than the first quartile in the interquartile range of the whole cohort), this was remeasured at a dilution that permitted interpolation on the standard curve. Control and DS samples were analyzed separately on four ELISA plates. The same two standard plasma samples were included on each plate in each assay to control for between‐plate variation; all inter‐assay CVs were < 15% (Table S1).

### 2.4 Data analysis and statistics

Data were analyzed by constructing a 7‐ to 10‐point standard curve using known concentrations of pure protein for each assay, interpolating the averaged optical density values for each sample on the curve, and multiplying the obtained values by the dilution factor. Data were plotted using GraphPad Prism 5, tested for normality using the Shapiro–Wilk test (alpha = 0.05), and analyzed statistically using Mann–Whitney tests or Pearson correlations as appropriate. Data were not adjusted for age or sex as no correlations were identified between these and protein concentrations. BMI was not available for controls.

## 3 RESULTS

### 3.1 Plasma levels of complement proteins are elevated in DS

**FIGURE 1:**
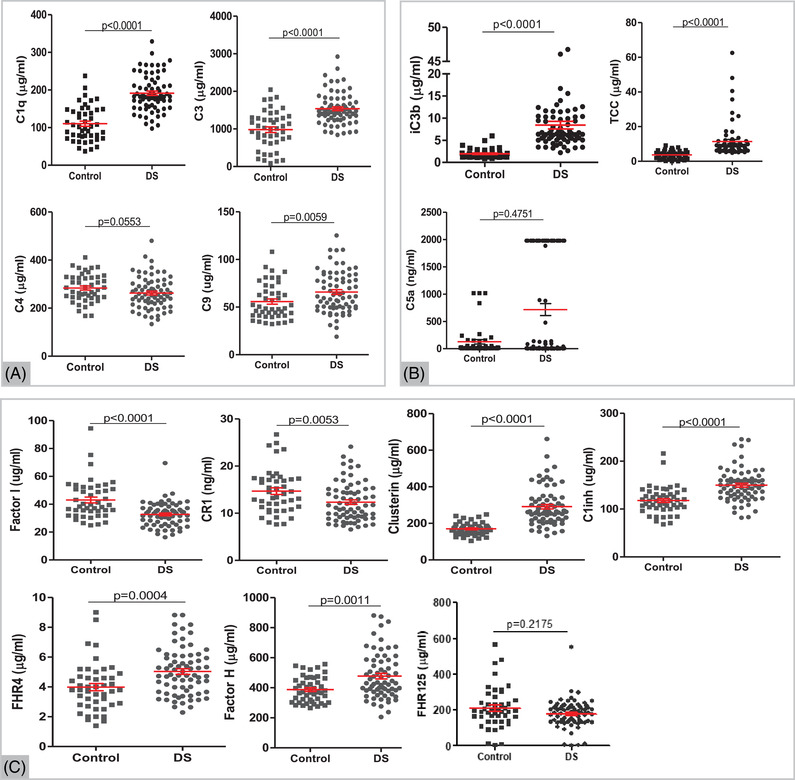
Complement components (A), activation products (B), and regulators (C) were measured in plasma from subjects with Down syndrome (DS) and healthy control individuals. Data are shown as scatter plots with mean ± standard error of the mean and were analyzed statistically using Mann–Whitney tests (*n* = 71 DS (68 for FH), 46 control)

**FIGURE 2:**
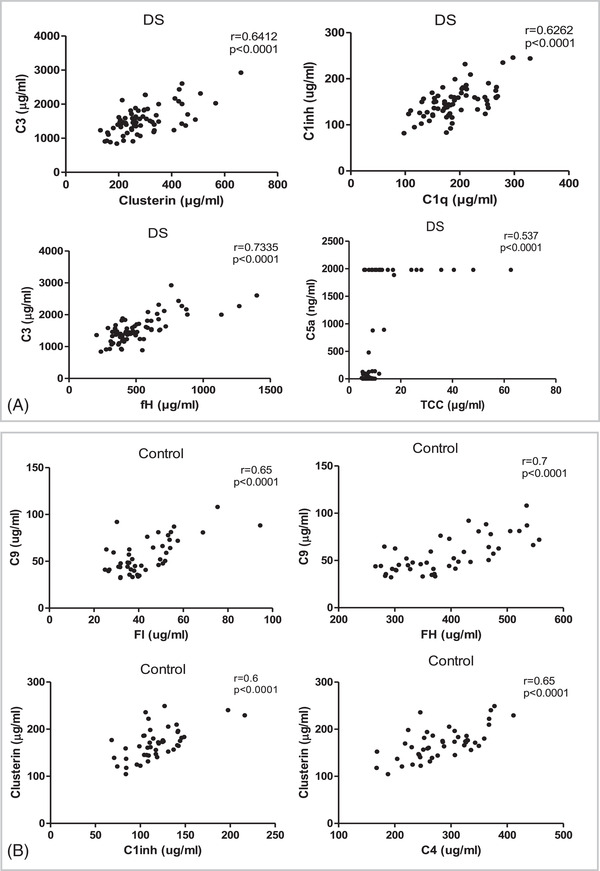
Correlation scores and *P*‐values between complement markers in the Down syndrome (DS) group (A) and control group (B)

Complement proteins were quantified by ELISA in DS and control subjects. Among the measured components, C1q, C3, and C9 were significantly elevated in DS compared to control (*p* < 0.05, Figure 1A). Two of the measured activation products TCC and iC3b, were also significantly increased in DS plasma (*p* < 0.001, Figure 1B). Plasma levels of the complement regulators clusterin, FH, C1 inhibitor, and FHR4 were significantly elevated in DS (*p* < 0.001), while FI and sCR1 were significantly decreased (*p* < 0.01, Figure 1C). There were no significant changes in levels of C4, C5a, or FHR125 in DS compared to controls. Within the DS group, several significant positive correlations were identified between complement proteins and are shown in Figure 2A (C3 and clusterin, FH; C1q and C1; TCC and C5a). Within the control group, a few significant positive correlations were identified between complement proteins and are shown in Figure 2B (C9 and FI, FH; clusterin and C4, C1 inhibitor). All other correlations are shown in Figure S1 in supporting information. Regarding demographic factors, C3 levels significantly correlated with BMI (Pearson r = 0.26, *p* < 0.05, Table S2), and FHR4 was significantly higher in women with DS, while TCC was significantly lower (Table S2).

### 3.2 Complement protein levels are altered in DS with AD

**FIGURE 3:**
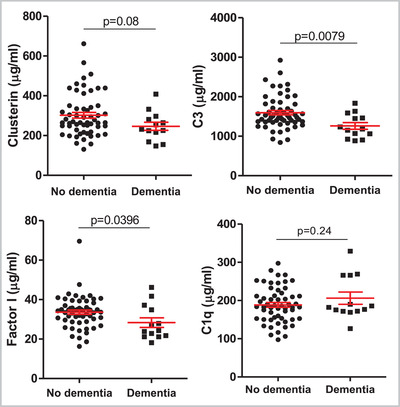
Plasma levels of clusterin, C3, FI, and C1q in Down syndrome with dementia (*n* = 13) versus no dementia (*n* = 57). Data are shown as mean ± standard error of the mean and were analyzed statistically using Mann–Whitney tests

Out of 71 individuals with DS, 13 were classified as having dementia at the time of sampling; as expected, the DS with AD group were significantly older. We tested whether any changes in complement protein levels occurred in DS with AD compared to DS without AD. Levels of C3 (*p* < 0.01) and FI (*p* < 0.05) were significantly decreased in DS with AD (Figure 3). No other significant differences in levels of complement biomarkers between the groups were identified. Clusterin levels were reduced and C1q levels increased in DS with AD but these did not reach significance (Figure 3).

### 3.3 The rs6656401 SNP is associated with increased sCR1 levels in DS

**FIGURE 4:**
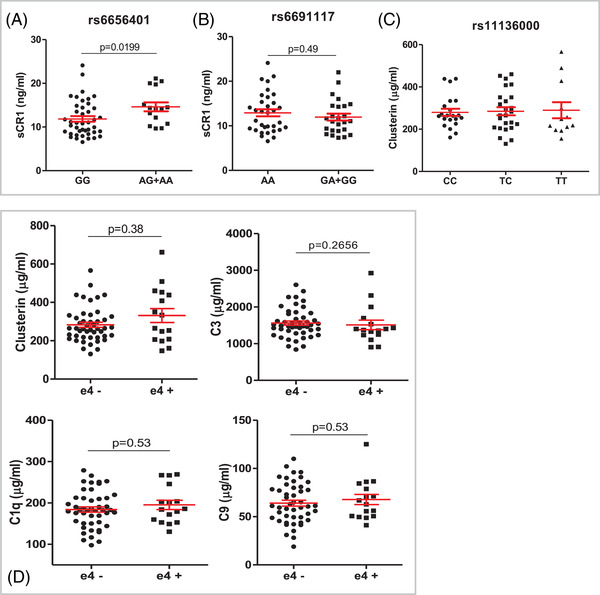
Effects of SNPs in CLU and CR1 on plasma levels of clusterin and sCR1 (A, B, C) and effects of *APOE* genotype on clusterin, C3, C1q, and C9 (D). Carriers of the A allele at rs6656401 had significantly higher CR1 levels compared to homozygotes for the G allele (A). There was no significant effect of rs6691117 or rs11136000 on plasma levels of sCR1 or clusterin, respectively (B, C). No significant effects of *APOE* ε4 genotype were observed on plasma levels of complement proteins (*p* = 0.8, D). Data are shown as mean ± SEM and were analyzed statistically using two‐tailed unpaired *t*‐tests, Mann–Whitney, or Kruskal–Wallis tests. *APOE*, apolipoprotein E; sCR1, soluble complement receptor 1; SEM, standard error of the mean; SNP, single nucleotide polymorphism

Plasma levels of sCR1 were compared between DS homozygous for the major allele at *CR1* SNP rs6656401 (G) and carriers of the minor allele (A); GG donors had significantly lower sCR1 levels in plasma compared to GA/AA donors (mean = 11.8 vs. 14.6 ng/ml, *p* < 0.05; Figure 4A). The *CR1* SNP rs669117 had no significant effect on plasma sCR1 levels (Figure 4B), and the *CLU* SNP rs1113600 was not associated with changes in plasma clusterin levels in the DS cohort (Figure 4C).

*APOE* genotyping was available on 63 of the DS subjects; from these we identified 16 heterozygotes for the ε4 allele, 2 of whom had AD. No significant changes in complement proteins were found in carriers of the ε4 allele compared to non‐carriers (Figure 4D).

## 4 DISCUSSION

Despite the high prevalence of immune dysregulation and chronic inflammatory diseases in DS, there are remarkably few studies investigating the role of complement, a key driver of inflammation. Some studies have shown the presence of complement proteins in DS brains;^15^ a few have explored levels of individual complement proteins in small numbers of DS plasma samples.^23^ We conducted an in‐depth analysis of components, regulators, and activation products encompassing the classical, alternative, and terminal pathways of complement in adults with DS versus euploid controls; further, we examined whether dementia or *APOE* ε4 genotype affected complement protein levels in DS.

The classical pathway, predominantly triggered by immune complexes, was first linked to DS in 1993; circulating C1q‐binding immune complexes were present in plasma from 70% of individuals with DS but only 10% of controls.^29^ The demonstration of C1q immunoreactivity with Aβ plaques in DS brains implicated complement in AD pathology in older adults with DS.^15^, ^18^ Studies of plasma proteome changes in children and adolescents with DS measured using mass spectrometry identified evidence of complement consumption, including decreased levels of C1q subunit A.^30^ In contrast, we found that plasma levels of C1q, the trigger for activation of the classical pathway, were significantly elevated in adults with DS compared to matched controls (mean 191 vs. 110 μg/ml). The reasons for this marked increase are unclear. C1q levels are modulated by inflammatory cytokines and plasma C1q levels are elevated in chronic infection states;^31^, ^32^ the observed increase may reflect an underlying inflammatory state in DS. C1q levels are known to increase with aging; accelerated aging in DS may also contribute to increased C1q plasma levels.^33^, ^34^ We also found that C3, the most abundant complement protein, and C9, essential for terminal pathway activity, were significantly increased in DS plasma; C3 is an acute phase reactant so elevated C3 may also be a consequence of inflammation. C3 levels are also increased in obesity, likely a consequence of obesity‐driven inflammation;^35^ notably, a recent study reported elevated C3 and C4 levels in adolescents with DS that strongly correlated with obesity.^23^ We also identified a significant positive correlation with BMI for plasma levels of C3, but not C4, in the DS group (Figure S1).

Regulatory mechanisms have evolved to prevent complement damage to self‐cells during responses to foreign pathogens or other activators.^36^ We quantified plasma levels of C1 inhibitor, FI, sCR1, clusterin, FH, and FH‐related proteins and found striking differences between the DS and control groups. C1 inhibitor, a critical regulator of the classical pathway that inactivates the C1 complex, was significantly elevated in DS compared to controls (mean 150 vs. 118 μg/ml) and correlated positively with C1q levels. Interestingly, higher C1 inhibitor levels were also reported in plasma from mothers carrying fetuses with DS compared to carriers of normal fetuses.^37^ Plasma levels of clusterin, a fluid‐phase regulator of the terminal pathway, were significantly higher in DS compared to control (292 vs. 172 μg/ml). While clusterin is an inhibitor of membrane attack complex formation, it has numerous other functions, including an important role in transporting cholesterol and regulating lipid homeostasis; elevated levels in DS might therefore have multiple effects on health and homeostasis. Notably, several biomarker studies in AD have implicated elevated plasma clusterin as a biomarker for late‐onset AD.^20^, ^38^ The *CLU* gene has also been implicated in AD through GWAS^21^, ^39^ with the minor T allele at rs11136000 associated with better cognitive function.^24^ In the DS cohort, this *CLU* SNP was not associated with a significant difference in clusterin plasma levels. FH is a plasma regulator of the alternative pathway C3 convertase while FHR4 is a FH‐related plasma protein that controls the activity of FH; levels of both were significantly elevated in DS plasma, with female subjects showing significantly higher levels compared to males in the DS group. In contrast, another study found increased FH levels in brain, liver, and spleen tissue in subjects with DS; however, this involved *post mortem* tissue from five subjects considerably older than our cohort.^40^ Levels of FI, an enzyme that provides powerful regulation of complement by inactivating complement convertases, were significantly decreased in DS plasma. Taken together, these findings support a substantial dysregulation of complement, primarily impacting the alternative pathway convertases. CR1 is a cell surface receptor for the activation fragments C3b and C4b; CR1 is cleaved from expressing cells under some circumstances and the released sCR1 can be measured in plasma. Elevated plasma levels of sCR1 have been reported in AD and suggested to have functional relevance.^41^ In our study sCR1 levels were significantly reduced in DS plasma compared to controls. Genetic variation in *CR1* has been shown to significantly contribute to AD risk and influence plasma sCR1 levels.^21^ The minor allele at rs6656401 was associated with expression of the long form of *CR1*,^22^ more rapid decline of cognitive function,^42^ and increased sCR1 plasma levels.^43^ Despite the observed decreased sCR1 levels in DS compared to controls, within the DS group we replicated the finding of increased sCR1 in carriers of the minor allele at rs6656401. In contrast, there was no significant change in plasma sCR1 in carriers of the minor allele of the *CR1* SNP at rs6691117, a missense variant (Ile2065Val) shown to be associated with decreased brain volume in mild cognitive impairment subjects.^44^ *APOE* ε4 genotype had no effect on plasma levels of complement proteins implicated in AD (C1q, C3, C9, clusterin) or any of the proteins measured; however, the number of *APOE* ε4 carriers in the sample sets was low (*n* = 16).

Complement activation products provide a direct indicator of ongoing complement dysregulation. We measured plasma levels of iC3b, C5a, and TCC, markers of activation of the C3 convertase, C5 convertase and terminal pathway, respectively. We found significantly elevated TCC and iC3b in DS compared to controls; however, the data showed that a subset of individuals with DS had very high levels of the activation markers TCC and C5a, suggesting that these individuals were profoundly dysregulated. Although we were not able to identify why this DS subset had such high levels of complement activation, we note that DS is often associated with autoimmune comorbidities and chronic inflammation that might be responsible for the observed complement dysregulation. Additionally, TCC levels were significantly elevated in men with DS compared to women, suggesting higher complement activity in male subjects.

Dementia resembling AD develops in most people with DS after the age of 40. In our cohort, 13 of 71 subjects showed signs of dementia when assessed clinically; this subgroup was significantly older than the non‐demented subjects (54 vs. 38 years, *p* < 0.0001). Comparison of complement proteins between the two subgroups showed that C3 and FI were significantly lower in the dementia group, though whether this is cause or consequence of the pathology is unclear. A recent report described significantly decreased CSF C3 levels in AD patients positive for Aβ, tau, and neurodegeneration markers.^45^ Others have reported association of low plasma C3 levels with increased risk for AD, particularly in *APOE* ε4/ε4 carriers.^46^ These findings suggest that AD pathology consumes C3 in the AD brain resulting in lower plasma levels; the demonstration that C3‐deficient APP transgenic mice displayed increased Aβ burden at 17 months compared to C3‐sufficient APP mice supports this mechanism.^47^ Additionally, C3 processing in the periphery may be accelerated by AD‐related comorbidities. Low levels of FI, linked to specific SNPs in the *FI* gene, are strongly associated with risk of age‐related macular degeneration (AMD).^48^ The association of low plasma FI with AMD has been ascribed to over‐activation of complement in the retina; although we did not test the *FI* SNP as a cause of low FI in the DS cohort, the same pro‐inflammatory consequences are likely. Clusterin has been reported as a biomarker for late‐onset AD in several studies;^49^, ^50^ however, there was no significant difference in clusterin levels in demented compared to non‐demented subjects with DS. Notably, while biomarker studies have implicated clusterin as a late‐onset sporadic AD biomarker, dementia in DS is an early onset genetically determined type of AD; no published studies address clusterin levels in familial AD.

## 5 CONCLUSIONS

Our study demonstrates that complement is dysregulated in DS. Plasma levels of activation products and key components were significantly elevated compared to controls; regulators showed significant changes compatible with a failure of regulation in the activation and terminal pathways. Subjects with DS and AD showed a distinct complement profile compared to non‐AD individuals, prompting further studies into complement system involvement in DS and the development of AD. While complement biomarkers provide clear evidence of immune dysregulation, they may not be sufficient to differentiate individuals with DS who will rapidly progress to dementia; they may, however, complement AD‐specific biomarkers to enhance risk prediction or early diagnosis of dementia in people with DS.

## CONFLICTS OF INTEREST

The authors report no conflicts of interest. Author disclosures are available in the supporting information.

## Supporting information



SUPPORTING INFORMATION

SUPPORTING INFORMATION

SUPPORTING INFORMATION

SUPPORTING INFORMATION
