# Supplementary material for: Complement dysregulation and Alzheimer's disease in Down syndrome
Source: Alzheimers Dement. 2022 Sep 23;19(4):1383–92. doi: 10.1002/alz.12799 (PMC10798358; doi:10.1002/alz.12799)
Supplement: Supplementary file 1 — SUPPORTING INFORMATION [file ALZ-19-1383-s001.pdf]

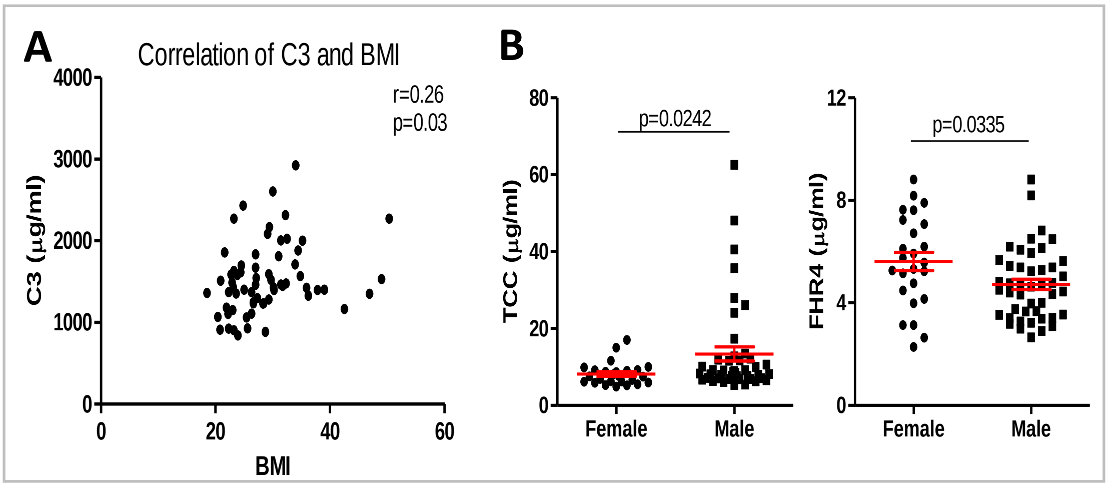

Supplementary Figure 1. Correlations and gender differences in DS. A. BMI and C3 levels were significantly correlated in the DS group (Pearson r=0.26, p=0.03). **B.** Male subjects with DS had significantly higher TCC and significantly lower FHR4 plasma levels compared to females.
