# Supplementary material for: Complement dysregulation and Alzheimer's disease in Down syndrome
Source: Alzheimers Dement. 2022 Sep 23;19(4):1383–92. doi: 10.1002/alz.12799 (PMC10798358; doi:10.1002/alz.12799)
Supplement: Supplementary file 2 — SUPPORTING INFORMATION [file ALZ-19-1383-s002.pdf]

Supplementary Table 1. Antibodies, proteins, and sample dilutions used in ELISA. All antibodies and standard proteins were produced in-house unless otherwise specified for each assay. Hycult: Hycult Biotech; Comptech: Complement Technology, Inc. mAb=monoclonal antibody raised in mouse.

**Biomarker**	**Capture antibody (concentration used, µg/ml)**	**Detection antibody (concentration used, µg/ml)**	**Standard protein**	**Plasma dilution**	**Lower/upper limit of detection (ng/ml)**	**Intra-/Inter-assay CV (%)**
C1q	mAb 9H10 (2)	Rabbit α-C1q (1)	C1q	1:16000	16-1000	5.6/11.5
C1 inhibitor	mAb anti-C1 inhibitor (2)	Rabbit anti-C1 inhibitor (1)	C1 inhibitor	1:16000	0.1-100	3.9/12.8
iC3b	mAb Clone 9 (5)	mAb bH6-HRP (Hycult) (2)	iC3b (Comptech)	1:50	32-5000	3.9/9.9
C3	Rabbit α-C3 (5)	Rabbit anti-C3-HRP (2)	C3 (Comptech)	1:20000	16-1000	6.4/10.5
C4	Rabbit anti-C4 (5)	mAb anti-C4-HRP (2)	C4 (Comptech)	1:4000	16-1000	2.8/9.8
C5a	mAb 2952 (Hycult)	mAb 295009-biotin (R&D systems)	C5a (Hycult)	1:2 – 1:50	15-2000	3.5/12.4
C9	mAb B7 (2)	Rabbit anti-C9-HRP (2)	C9	1:1000	1-200	3.6/1
CR1	Rabbit α-CR1 (2.5)	mAb MBI35 (1)	CR1	1:5	2-100	3.7/10.3
TCC	mAb aE11 (Hycult) (5)	E2-biotin	TCC	1:50	5-5000	5.4/9.2
FI	mAb 7B5 (2)	Rabbit anti-FI (1)	Factor I (Comptech)	1:200	63-1000	8.5/14
FH	mAb OX-24 (2)	mAb 35H9 (2)	Factor H	1:2000	1.5-1500	3.9/13.4
FHR4	mAb 4E9 (2)	mAb Clone 150-HRP (1)	FHR4	1:50	2-1000	4/3
FHR125	mAb MBI125 (5)	mAb 35H9 (5)	FHR125	1:500	4-5000	4.1/5.5
Clusterin	mAb 2D5 (5)	mAb 4C7-HRP (2)	Clusterin	1:2000	8-2000	4/10.5
