# Supplementary material for: Complement dysregulation and Alzheimer's disease in Down syndrome
Source: Alzheimers Dement. 2022 Sep 23;19(4):1383–92. doi: 10.1002/alz.12799 (PMC10798358; doi:10.1002/alz.12799)
Supplement: Supplementary file 3 — SUPPORTING INFORMATION [file ALZ-19-1383-s003.pdf]

A								Correlations							
		Clusterin (µg/ml)	FH (µg/ml)	C9 (µg/ml)	TCC (µg/ml)	C1 inh (µg/ml)	C5a (µg/ml)	FHR4 (µg/ml)	iC3b (µg/ml)	FHR125 (µg/ml)	C3 (µg/ml)	FI (µg/ml)	CR1 (ng/ml)	C1q (µg/ml)	C4 (µg/ml)
Clusterin (µg/ml)	Pearson Correlation	1	.453**	-0.107	-0.015	0.205	0.070	0.155	-0.055	.301*	.667**	0.128	-.316**	0.014	0.063
	Sig. (2-tailed)		0.000	0.374	0.901	0.086	0.563	0.196	0.649	0.011	0.000	0.289	0.007	0.910	0.601
	N	71	71	71	70	71	71	71	71	71	71	71	71	71	71
FH (µg/ml)	Pearson Correlation	.453**	1	.313**	-0.152	0.230	-0.014	0.053	-0.041	-0.044	.730**	.354**	0.159	0.223	0.217
	Sig. (2-tailed)	0.000		0.008	0.208	0.054	0.911	0.659	0.736	0.713	0.000	0.002	0.185	0.062	0.070
	N	71	71	71	70	71	71	71	71	71	71	71	71	71	71
C9 (µg/ml)	Pearson Correlation	-0.107	.313**	1	0.141	.382**	-0.036	.368**	0.067	-0.075	0.133	0.213	0.142	.454**	0.205
	Sig. (2-tailed)	0.374	0.008		0.243	0.001	0.768	0.002	0.581	0.537	0.269	0.075	0.239	0.000	0.087
	N	71	71	71	70	71	71	71	71	71	71	71	71	71	71
TCC (µg/ml)	Pearson Correlation	-0.015	-0.152	0.141	1	0.223	.537**	0.077	-0.049	0.226	-0.177	-0.015	-0.032	0.124	-0.022
	Sig. (2-tailed)	0.901	0.208	0.243		0.064	0.000	0.526	0.690	0.060	0.143	0.902	0.795	0.307	0.856
	N	70	70	70	70	70	70	70	70	70	70	70	70	70	70
C1 inh (µg/ml)	Pearson Correlation	0.205	0.230	.382**	0.223	1	0.027	0.095	-0.006	0.071	0.206	.307**	0.056	.626**	0.040
	Sig. (2-tailed)	0.086	0.054	0.001	0.064		0.826	0.433	0.960	0.556	0.085	0.009	0.644	0.000	0.739
	N	71	71	71	70	71	71	71	71	71	71	71	71	71	71
C5a (µg/ml)	Pearson Correlation	0.070	-0.014	-0.036	.537**	0.027	1	-0.031	0.118	0.085	-0.116	0.030	0.092	-0.093	-0.006
	Sig. (2-tailed)	0.563	0.911	0.768	0.000	0.826		0.798	0.327	0.480	0.335	0.804	0.446	0.441	0.958
	N	71	71	71	70	71	71	71	71	71	71	71	71	71	71
FHR4 (µg/ml)	Pearson Correlation	0.155	0.053	.368**	0.077	0.095	-0.031	1	0.214	.371**	0.156	-0.086	-0.135	0.067	0.195
	Sig. (2-tailed)	0.196	0.659	0.002	0.526	0.433	0.798		0.073	0.001	0.193	0.477	0.263	0.576	0.104
	N	71	71	71	70	71	71	71	71	71	71	71	71	71	71
iC3b (µg/ml)	Pearson Correlation	-0.055	-0.041	0.067	-0.049	-0.006	0.118	0.214	1	0.052	0.015	.242*	-0.176	-0.113	-0.013
	Sig. (2-tailed)	0.649	0.736	0.581	0.690	0.960	0.327	0.073		0.665	0.898	0.042	0.141	0.348	0.916
	N	71	71	71	70	71	71	71	71	71	71	71	71	71	71
FHR125 (µg/ml)	Pearson Correlation	.301*	-0.044	-0.075	0.226	0.071	0.085	.371**	0.052	1	0.061	0.109	-.329**	-0.044	0.147
	Sig. (2-tailed)	0.011	0.713	0.537	0.060	0.556	0.480	0.001	0.665		0.614	0.368	0.005	0.717	0.221
	N	71	71	71	70	71	71	71	71	71	71	71	71	71	71
C3 (µg/ml)	Pearson Correlation	.667**	.730**	0.133	-0.177	0.206	-0.116	0.156	0.015	0.061	1	.303*	-0.046	0.108	0.043
	Sig. (2-tailed)	0.000	0.000	0.269	0.143	0.085	0.335	0.193	0.898	0.614		0.010	0.700	0.369	0.722
	N	71	71	71	70	71	71	71	71	71	71	71	71	71	71
FI (µg/ml)	Pearson Correlation	0.128	.354**	0.213	-0.015	.307**	0.030	-0.086	.242*	0.109	.303*	1	0.062	0.188	-0.029
	Sig. (2-tailed)	0.289	0.002	0.075	0.902	0.009	0.804	0.477	0.042	0.368	0.010		0.608	0.116	0.808
	N	71	71	71	70	71	71	71	71	71	71	71	71	71	71
CR1 (ng/ml)	Pearson Correlation	-.316**	0.159	0.142	-0.032	0.056	0.092	-0.135	-0.176	-.329**	-0.046	0.062	1	0.110	-0.119
	Sig. (2-tailed)	0.007	0.185	0.239	0.795	0.644	0.446	0.263	0.141	0.005	0.700	0.608		0.362	0.324
	N	71	71	71	70	71	71	71	71	71	71	71	71	71	71
C1q (µg/ml)	Pearson Correlation	0.014	0.223	.454**	0.124	.626**	-0.093	0.067	-0.113	-0.044	0.108	0.188	0.110	1	0.193
	Sig. (2-tailed)	0.910	0.062	0.000	0.307	0.000	0.441	0.576	0.348	0.717	0.369	0.116	0.362		0.106
	N	71	71	71	70	71	71	71	71	71	71	71	71	71	71
C4 (µg/ml)	Pearson Correlation	0.063	0.217	0.205	-0.022	0.040	-0.006	0.195	-0.013	0.147	0.043	-0.029	-0.119	0.193	1
	Sig. (2-tailed)	0.601	0.070	0.087	0.856	0.739	0.958	0.104	0.916	0.221	0.722	0.808	0.324	0.106	
	N	71	71	71	70	71	71	71	71	71	71	71	71	71	71

*. Correlation is significant at the 0.05 level (2-tailed).

**. Correlation is significant at the 0.01 level (2-tailed).

B

Correlations

		C1inh (ug/ml)	C1q (ug/ml)	FHR4 (ug/ml)	Clusterin (ug/ml)	TCC (ug/ml)	FI (ug/ml)	C9 (ug/ml)	C3 (ug/ml)	C4 (ug/ml)	FHR125 (ug/ml)	FH (ug/ml)	iC3b (ug/ml)	C5a (ng/ml)	CR1 (ng/ml)
C1inh (ug/ml)	Pearson Correlation	1	.500**	0.199	.595**	0.000	.339*	.580**	-0.265	.513**	0.159	.508**	.460**	-0.197	0.146
	Sig. (2-tailed)		0.000	0.185	0.000	0.998	0.023	0.000	0.078	0.000	0.292	0.000	0.001	0.189	0.333
	N	46	46	46	46	46	45	45	45	46	46	46	46	46	46
C1q (ug/ml)	Pearson Correlation	.500**	1	0.238	0.182	-0.180	0.011	0.217	-.383**	0.211	0.144	.309*	.320*	-0.188	.333*
	Sig. (2-tailed)	0.000		0.111	0.227	0.230	0.944	0.152	0.009	0.160	0.341	0.037	0.030	0.210	0.024
	N	46	46	46	46	46	45	45	45	46	46	46	46	46	46
FHR4 (ug/ml)	Pearson Correlation	0.199	0.238	1	0.151	.348*	.456**	.489**	-0.079	.348*	.319*	.453**	-0.148	-0.124	.419**
	Sig. (2-tailed)	0.185	0.111		0.315	0.018	0.002	0.001	0.604	0.018	0.031	0.002	0.326	0.413	0.004
	N	46	46	46	46	46	45	45	45	46	46	46	46	46	46
Clusterin (ug/ml)	Pearson Correlation	.595**	0.182	0.151	1	0.274	.527**	.588**	-0.068	.651**	0.198	.549**	0.243	-0.134	-0.015
	Sig. (2-tailed)	0.000	0.227	0.315		0.065	0.000	0.000	0.655	0.000	0.188	0.000	0.103	0.373	0.923
	N	46	46	46	46	46	45	45	45	46	46	46	46	46	46
TCC (ug/ml)	Pearson Correlation	0.000	-0.180	.348*	0.274	1	.478**	.322*	0.261	0.175	0.194	.376*	-0.290	0.180	-0.032
	Sig. (2-tailed)	0.998	0.230	0.018	0.065		0.001	0.031	0.084	0.243	0.196	0.010	0.051	0.230	0.833
	N	46	46	46	46	46	45	45	45	46	46	46	46	46	46
FI (ug/ml)	Pearson Correlation	.339*	0.011	.456**	.527**	.478**	1	.655**	0.194	.459**	0.121	.565**	-0.064	-0.125	0.163
	Sig. (2-tailed)	0.023	0.944	0.002	0.000	0.001		0.000	0.208	0.001	0.430	0.000	0.674	0.413	0.284
	N	45	45	45	45	45	45	45	44	45	45	45	45	45	45
C9 (ug/ml)	Pearson Correlation	.580**	0.217	.489**	.588**	.322*	.655**	1	-0.249	.539**	.314*	.701**	0.223	-0.108	0.173
	Sig. (2-tailed)	0.000	0.152	0.001	0.000	0.031	0.000		0.103	0.000	0.036	0.000	0.141	0.481	0.257
	N	45	45	45	45	45	45	45	44	45	45	45	45	45	45
C3 (ug/ml)	Pearson Correlation	-0.265	-.383**	-0.079	-0.068	0.261	0.194	-0.249	1	-0.083	-.313*	-0.012	-0.083	-0.032	-0.109
	Sig. (2-tailed)	0.078	0.009	0.604	0.655	0.084	0.208	0.103		0.587	0.036	0.936	0.588	0.834	0.478
	N	45	45	45	45	45	44	44	45	45	45	45	45	45	45
C4 (ug/ml)	Pearson Correlation	.513**	0.211	.348*	.651**	0.175	.459**	.539**	-0.083	1	-0.106	.539**	0.249	-0.191	0.133
	Sig. (2-tailed)	0.000	0.160	0.018	0.000	0.243	0.001	0.000	0.587		0.482	0.000	0.095	0.204	0.380
	N	46	46	46	46	46	45	45	45	46	46	46	46	46	46
FHR125 (ug/ml)	Pearson Correlation	0.159	0.144	.319*	0.198	0.194	0.121	.314*	-.313*	-0.106	1	0.050	-0.218	-0.049	-0.061
	Sig. (2-tailed)	0.292	0.341	0.031	0.188	0.196	0.430	0.036	0.036	0.482		0.741	0.146	0.745	0.687
	N	46	46	46	46	46	45	45	45	46	46	46	46	46	46
FH (ug/ml)	Pearson Correlation	.508**	.309*	.453**	.549**	.376*	.565**	.701**	-0.012	.539**	0.050	1	0.199	0.004	0.227
	Sig. (2-tailed)	0.000	0.037	0.002	0.000	0.010	0.000	0.000	0.936	0.000	0.741		0.184	0.981	0.129
	N	46	46	46	46	46	45	45	45	46	46	46	46	46	46
iC3b (ug/ml)	Pearson Correlation	.460**	.320*	-0.148	0.243	-0.290	-0.064	0.223	-0.083	0.249	-0.218	0.199	1	-0.244	0.265
	Sig. (2-tailed)	0.001	0.030	0.326	0.103	0.051	0.674	0.141	0.588	0.095	0.146	0.184		0.102	0.075
	N	46	46	46	46	46	45	45	45	46	46	46	46	46	46
C5a (ng/ml)	Pearson Correlation	-0.197	-0.188	-0.124	-0.134	0.180	-0.125	-0.108	-0.032	-0.191	-0.049	0.004	-0.244	1	-0.143
	Sig. (2-tailed)	0.189	0.210	0.413	0.373	0.230	0.413	0.481	0.834	0.204	0.745	0.981	0.102		0.344
	N	46	46	46	46	46	45	45	45	46	46	46	46	46	46
CR1 (ng/ml)	Pearson Correlation	0.146	.333*	.419**	-0.015	-0.032	0.163	0.173	-0.109	0.133	-0.061	0.227	0.265	-0.143	1
	Sig. (2-tailed)	0.333	0.024	0.004	0.923	0.833	0.284	0.257	0.478	0.380	0.687	0.129	0.075	0.344	
	N	46	46	46	46	46	45	45	45	46	46	46	46	46	46

**. Correlation is significant at the 0.01 level (2-tailed).

*. Correlation is significant at the 0.05 level (2-tailed).

Supplementary Figure 1. Pearson correlation scores and P-values for correlations between complement proteins within the DS group (**A**) and control group (**B**).
